# Deep Learning and Hyperspectral Images Based Tomato Soluble Solids Content and Firmness Estimation

**DOI:** 10.3389/fpls.2022.860656

**Published:** 2022-05-02

**Authors:** Yun Xiang, Qijun Chen, Zhongjing Su, Lu Zhang, Zuohui Chen, Guozhi Zhou, Zhuping Yao, Qi Xuan, Yuan Cheng

**Affiliations:** ^1^Institute of Cyberspace Security, Zhejiang University of Technology, Hangzhou, China; ^2^Institute of Vegetables, Zhejiang Academy of Agricultural Sciences, Hangzhou, China

**Keywords:** hyperspectral imaging, deep learning, cherry tomato, soluble solids content, firmness, one-dimensional convolutional neural networks

## Abstract

Cherry tomato (*Solanum lycopersicum*) is popular with consumers over the world due to its special flavor. Soluble solids content (SSC) and firmness are two key metrics for evaluating the product qualities. In this work, we develop non-destructive testing techniques for SSC and fruit firmness based on hyperspectral images and the corresponding deep learning regression model. Hyperspectral reflectance images of over 200 tomato fruits are derived with the spectrum ranging from 400 to 1,000 nm. The acquired hyperspectral images are corrected and the spectral information are extracted. A novel one-dimensional (1D) convolutional ResNet (Con1dResNet) based regression model is proposed and compared with the state of art techniques. Experimental results show that, with a relatively large number of samples our technique is 26.4% better than state of art technique for SSC and 33.7% for firmness. The results of this study indicate the application potential of hyperspectral imaging technique in the SSC and firmness detection, which provides a new option for non-destructive testing of cherry tomato fruit quality in the future.

## 1. Introduction

Tomato is a very popular fruit globally and its annual production reaches 186.82 million tons in 2020 (FAO, 2021). Tomatoes contain rich nutrients such as lycopene, β-carotene and vitamins (Sainju et al., 2003; Gao et al., 2020) etc. To facilitate the tomato production, processing, and marketing, its grade and maturity needs to be evaluated. In general, soluble solids and firmness are two key indicators (Beckles, 2012). SSC can be used to grade tomato quality and the firmness can be used to determine fruit maturity (Peng and Lu, 2008). The existing measuring techniques relying upon chemistry reactions can derive the SSC value accurately. However, the destructive methods can not be applied in high volume measurements. Moreover, there are significant variations so that sampling can be inefficient and inaccurate (Li et al., 2013). Therefore, in this work, we propose a hyperspectral imaging and deep learning based technique to measure tomato SSC and firmness nondestructively, accurately, and in high volume.

Spectroscopy is a widely used nondestructive testing method for fruit inspection. It includes various imaging techniques including visible, near infrared, terahertz spectroscopy, raman spectroscopy, and hyperspectral imaging etc. Visible and near infrared spectroscopy are rapid, convenient, and low cost. However, they are contrained by limited spectral band (Yin et al., 2019). Terahertz (THz) radiation has microwave and infrared properties and is able to penetrate and interact with many common materials, its equipments are very expensive (Afsah-Hejri et al., 2019). Raman spectroscopy is easy to operate, quick to measure, and contains rich information. However, its performance is inferior in terms of stability and sensitivity (Weng et al., 2019). Hyperspectral imaging technology can simultaneously detect the two-dimensional spatial information and 1D spectral information, therefore combine image and spectral characteristics (Adão et al., 2017). It can derive the overall spatial spectral information of cherry tomato and thus, is selected as the imaging method.

Hyperspectral imaging has been widely used for non-destructive testing in various fields, such as detection of plant disease stress (Lowe et al., 2017), industrial food packaging (Medus et al., 2021), medical image classification (Jeyaraj and Nadar, 2019), and horticultural products (Huang et al., 2017). Hyperspectral images are also effective for quality analysis of fruits. Rahman et al. (2017) use hyperspectral imaging to estimate metrics such as water content and PH readings. Zhou et al. (2020) use it to classify the maize seeds. Fan et al. (2015) use it to predict SSC and firmness in pears. They combine the competitive adaptive reweighted sampling and successive projection algorithm to select the variables as in partial least squares regression (PLSR). Rahman et al. (2018) fit sweetness and firmness of tomato. Lu et al. (2017) gives a review of the application of recent hyperspectral techniques. Therefore, hyperspectral imaging techniques can effectively measure or classify fruit and vegetable products.

The existing spectral analysis techniques typically require a regression model to fit the spectral data (Jiang and Chen, 2015), which have been widely used in areas such as food, petrochemical, and pharmaceutical fields (Chen et al., 2018). In general, various machine learning based algorithms are employed to build classification and regression models for hyperspectral images. Li et al. (2016) use PLSR to build a hyperspectral regression model to predict the water status of grapevines. Guo C. et al. (2016) develop an SVM model to assess the maturity of strawberries. Abdulridha et al. (2019) combine hyperspectral imaging and KNN algorithm to differentiate ulcer-infected fruits. Ji et al. (2019) use the AdaBoost algorithm to recognize the rate of potato damage. The machine learning algorithms typically perform a filtering process on the spectral bands.

Deep learning models, e.g., convolutional neural network (CNN), can learn features automatically from a large amount of data (Guo Y. et al., 2016). It is widely used in medics (Esteva et al., 2019), industry (Hossain et al., 2018), agriculture (Kamilaris and Prenafeta-Boldú, 2018), object detection (Zou et al., 2019), and signal processing (Yu and Deng, 2010) etc. This technique is also used in building hyperspectral correction models for classification and prediction. Paoletti et al. (2019) summarize the application of deep learning for hyperspectral image classification and conclude that CNN based models are generally more effective due to their capacity to extract highly discriminatory features and leverage the spatial and spectral information. Qiu et al. (2018) demonstrate that CNN outperforms other machine learning methods for rice variety identification application. Kong et al. (2014) track activity of peroxidase in tomato hyperspectral images using genetic algorithm and extreme learning machine. Rahman et al. (2018) develop a regression model in 1,000–1,550 nm hyperspectral images using PLSR method to estimate sweetness and firmness with *R*^2^ of 0.672 and 0.548, respectively.

In this work, we propose a deep learning and hyperspectral imaging based technique to estimate the metrics inside cherry tomato. Specifically, we have made the following contributions.

1. We demonstrate the effectiveness of deep learning based techniques and propose such a model to estimate fruit SSC and firmness.
2. We explore the tradeoff between sample number and model accuracy.
3. We collect real-world field data and evaluate the performance of our technique.

The experimental results show that our technique is 26.4% better than the state of art technique in SSC estimation and 33.4% in firmness estimation.

## 2. Materials and Methods

In this section, we describe the sample preparation, hyperspectral image acquisition and calibration, and the ground truth measurements for SSC and firmness methods. Specifically, we develop Con1dResNet, a deep learning and hyperspectral image based SSC and firmness estimation technique. Meanwhile, four comparing baseline techniques are also introduced.

### 2.1. Sample Preparation

The sample plant is a local mainstream cherry tomato (cultivar: Zheyingfen-1), which is dominating in the local market more with 70% share. The seeds first grow in the lab with tight environment control for one month. Then the seedlings are transplanted to the greenhouse of the Zhejiang academy of agricultural sciences, Hangzhou, China (east longitude 120°2', north latitude 30°27') on April 2nd (early spring), 2021. Field management is implemented following the standard commercial procedures. Cherry tomato fruits are harvested in June 2021. Two-hundred fully mature fruits are collected from 50 different plants for hyperspectral image acquisition. Firmness and soluble solids content of each fruit is measured using portable firmness tester and hand-held refractometer after image acquisition, respectively. The fruits of “Zheyingfen-1” were ideal for our study due to its highly soluble solid content limit, which would help extending the modeling range in this study.

#### 2.1.1. Hyperspectral Image Acquisition

**Figure 1:**
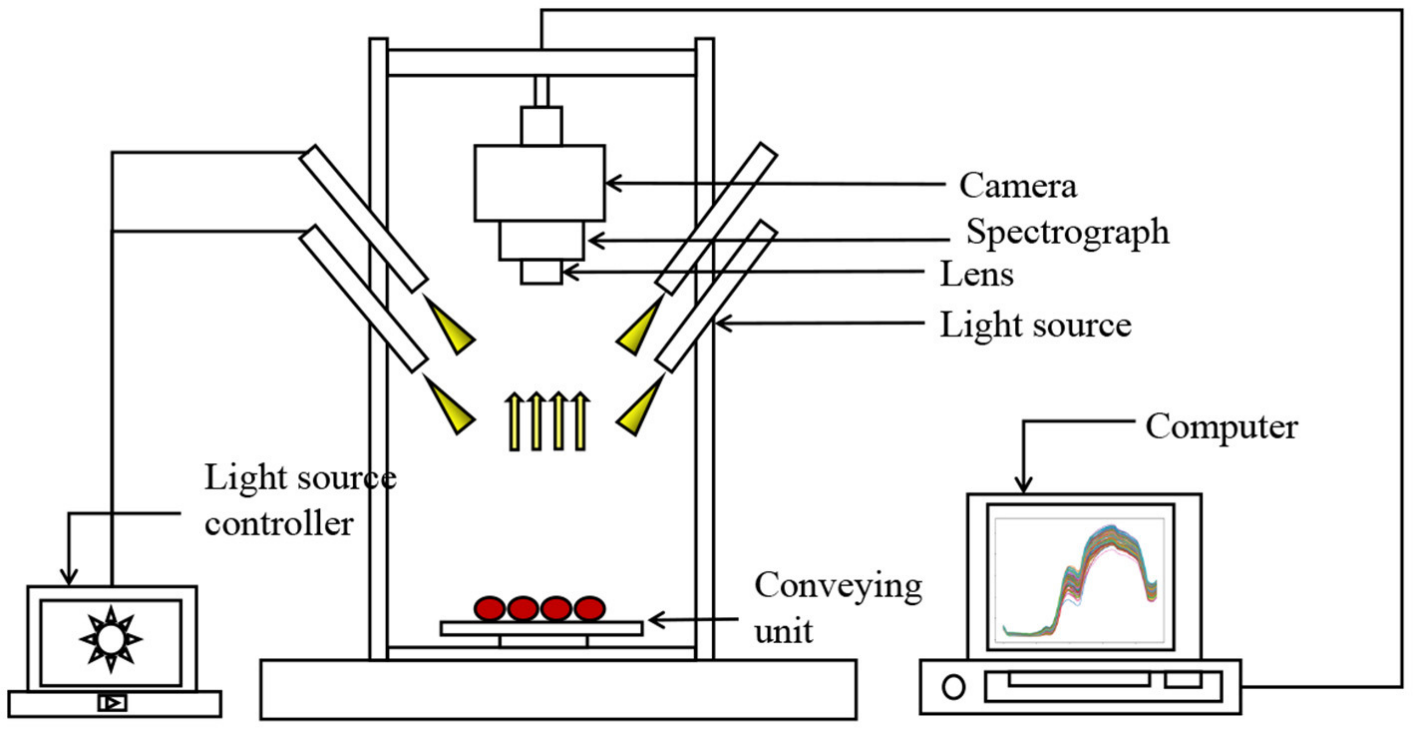
Schematic of the hyperspectral imaging system for acquiring spectral scattering images from cherry tomatoes.

**Figure 2:**
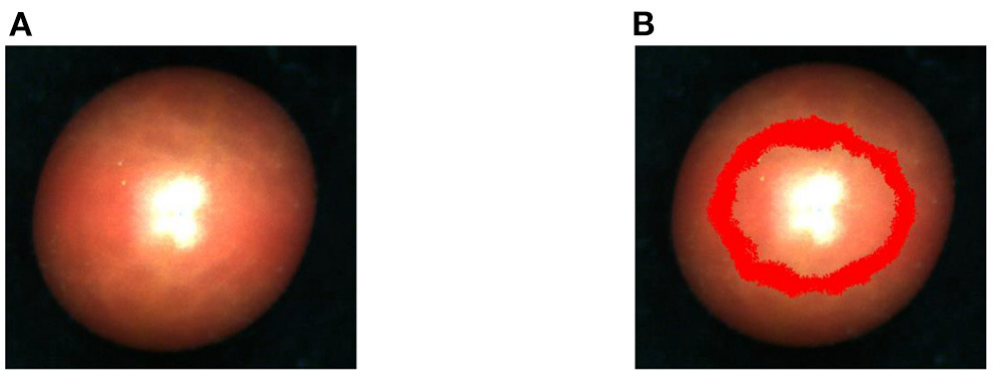
**(A)** ENVI original hyperspectral image. **(B)** Area map of ROI acquired by ENVI.

A hyperspectral imaging system is used to derive the clear and unblurred hyperspectral images as shown in Figure 1. We use a push-broom hyperspectral camera (PIKA XC, Resonon Inc., Bozeman, MT, USA) mounted 20 cm above the tomato samples. The hyperspectral images are acquired with the spatial resolution of 50 pixels per *mm*^2^ under artificial lighting (four 15 W 12 V light bulbs with two on either side of the lens). The main specifications of the hyperspectral camera were: interface, Firewire (IEEE 1394b), digital output (14 bit), and angular field of view of 7°. The objective lens had a 17 mm focal length (maximum aperture of F1.4), optimized for the hyperspectral. We acquire reflectance data in 462 spectral bands from 386 to 1,004 nm with a spectral resolution of 1.3 nm. Due to the convex surface of the samples, the uneven reflection creates a highlighted region near the vertical axial as shown in Figure 2A. Thus, we use ENVI5.3 (ITT, Visual Information Solutions, Boulder, CO, USA) (Su et al., 2021) to avoid the highlight region and extract the reflection value for each band from the region of interest (Xue, 2010; Fu et al., 2021; Figure 2B). The processed cherry tomato samples and the corresponding hyperspectral images are divided into training set, validation set, and test set with ratio of 7:1:2, respectively. We use varying dataset size, with a small set if 50 samples and a large set of 200 samples.

#### 2.1.2. Hyperspectral Image Calibration

**Figure 3:**
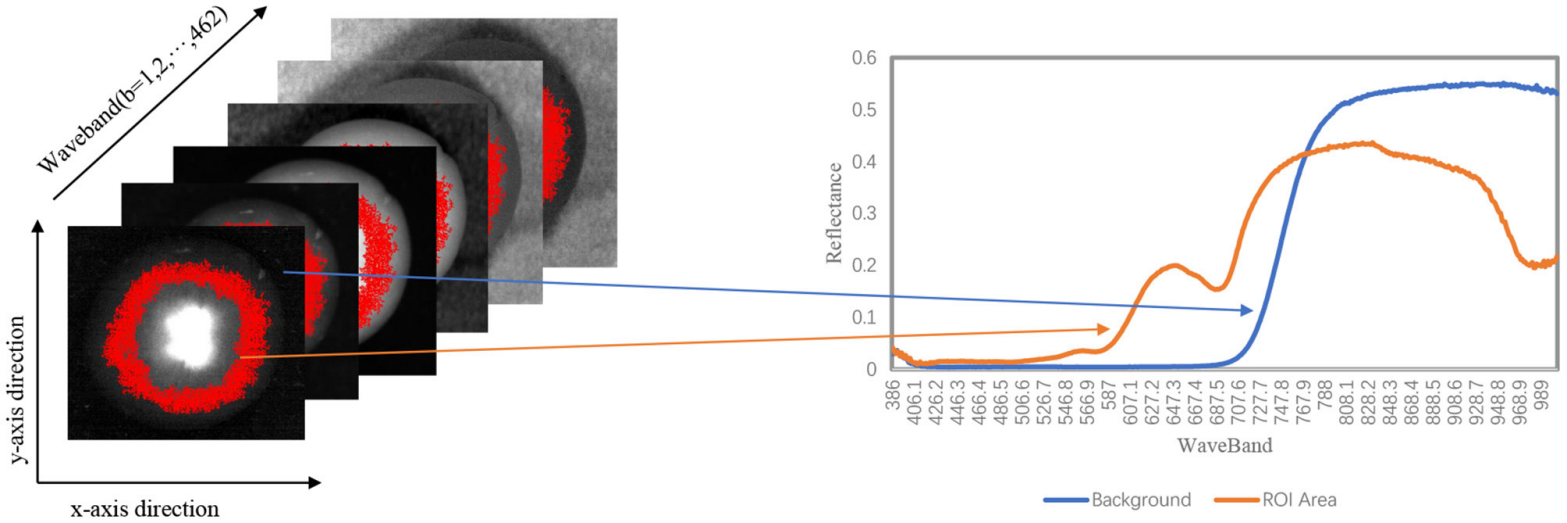
Schematic diagram of the structure and data of the corrected hyperspectral image: spatial axis x, y, and wavebands.

In reflectance calibration, the acquired hyperspectral image needs to be calibrated for the background spectral response of the instrument and the thermal dark current of the camera. The spectral data collected from the CCD device contains only the detector signal intensity value (Elmasry et al., 2012). Therefore, it is required to convert the raw data to reflectance or absorptivity values by comparing to the spectra of standard reference substances (Burger and Geladi, 2005) as shown in Figure 3. The reflectance can be derived using the following equation.


$$R_{c}=\frac{R_{ori}-R_{dark}}{R_{white}-R_{dark}},$$

where *R*_*c*_ is the corrected hyperspectral reflectance, *R*_*ori*_ is the original reflection value extracted from ENVI5.3, *R*_*dark*_ is the dark environment hyperspectral image reflection value, which is acquired using an opaque lens cap covering the hyperspectral lens, and *R*_*white*_ is the reflection value of a piece of white Teflon (100% reflectance, K-Mac Plastics, MI, USA).

#### 2.1.3. Baseline Measurement

The baseline firmness and SSC of cherry tomatoes are measured in the lab. For the firmness measurement, the cherry tomatoes are fixed on a portable firmness measurement equipment (GY-4, Zhejiang Top Cloud-Agri Technology Co., Ltd, China). The equipment is zero-calibrated. Starting from the contact of the probe with the cherry tomato surface, the 10 mm downward pressure is considered as the firmness value.

SSC measurements follow the firmness measurements. Cherry tomatoes are cut along the vertical axis and wrapped using a gauze. Then they are squeezed manually to force out the solution. About one milliliter tomato solution is placed on the prism of a portable digital refractometer (PAL-1, ATAGO CHINA Guangzhou Co., Ltd, China) to derive the baseline SSC readings. Each cherry tomato sample solution is measured for three times and the results are averaged to reduce the effect of random environment events.

### 2.2. Hyperspectral Pre-processing

#### 2.2.1. Multiple Scattering Correction

Multiple scattering correction (MSC) is a commonly used algorithm for hyperspectral data pre-processing (Zhang et al., 2012). MSC can effectively eliminate the spectral differences due to varying scattering levels, thus enhance the correlation between the spectrum and the data. This method can correct the baseline shifting and skewing using ideal spectra. The specific implementation is as follows:

1. assign the average of all hyperspectral data as “ideal spectrum;”
2. use one-dimensional linear regression and least square method to derive the baseline shifting and skewing values for each sample;
3. subtract the baseline shifting value and the divide the result using the skewing value to generate the corrected spectrum.

#### 2.2.2. Spectral Differential Techniques

The spectral differentiation technique involves mathematical simulation of the reflectance spectrum and calculation of differential values of different orders to determine the spectral bending point and the wavelength for the maximum and minimum reflectance. The data processed using second-order differentiation can reflect the spectrum variation caused by the absorption of biochemical elements such as plant chlorophyll, water, and nitrogen (Liu, 2020).

### 2.3. Image Processing Models

#### 2.3.1. Deep Learning Model

Deep learning models are widely used in medical image processings (Kiranyaz et al., 2015). However, in this work, it is required to build appropriate regression models. In general, we propose the Con1dResNet model to estimate the tomato SSC and firmness.

ResNet (He et al., 2016), a popular model for image classification, can solve the degradation problem of deep networks. Thus, ResNet34 is implemented as the baseline network structure, and the original convolutional layer is reconstructed to be one-dimensional, accordingly. We use the Adam optimizer and mean squared error loss function. We change the number of categories output by the last fully connected layer to one so that the network directly outputs the estimated values of SSC and firmness.

**Figure 4:**
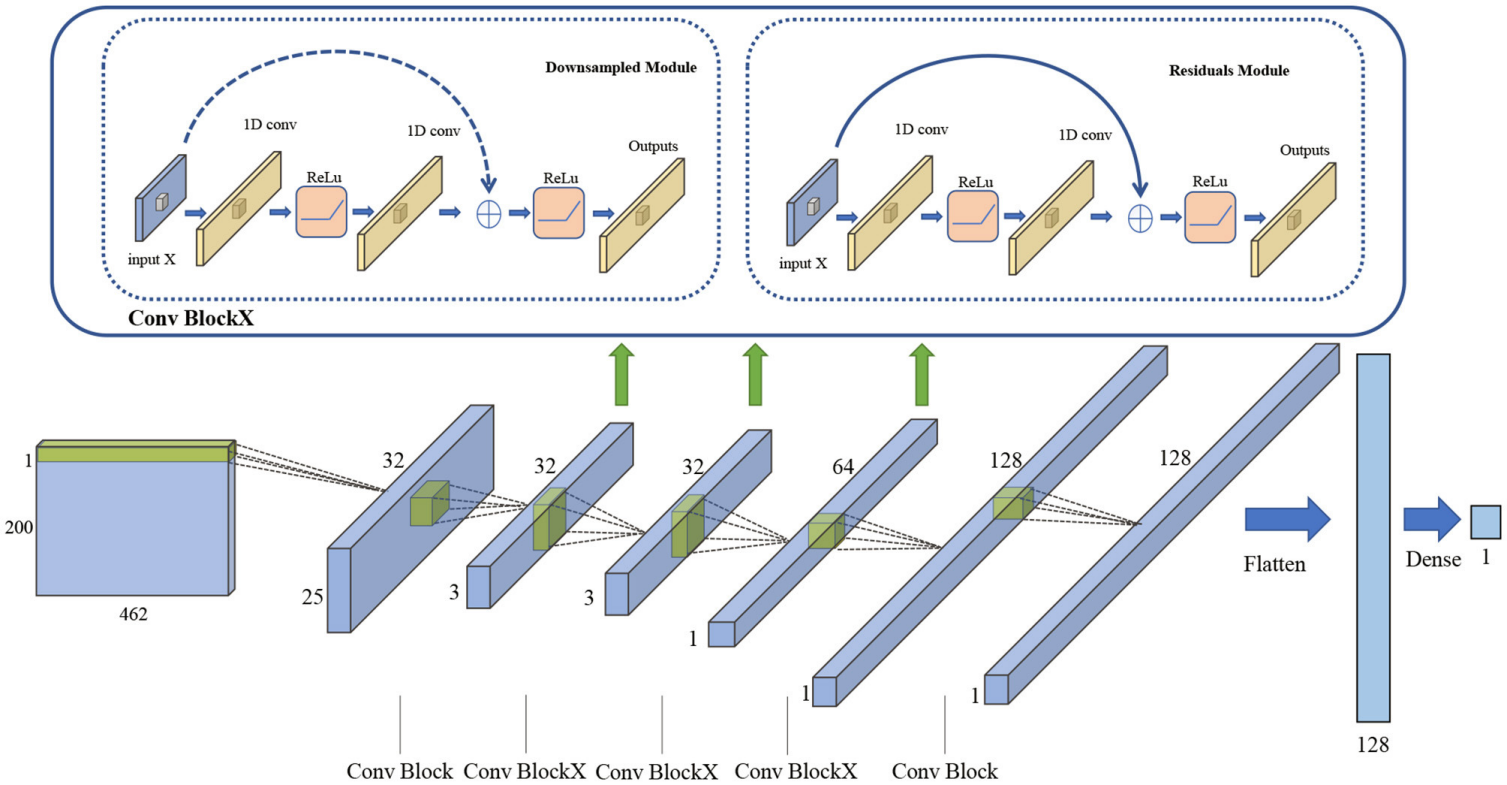
Con1dResNet network structure schematic.

The specific network structure is shown in Figure 4. In the figure, the input is the reflectance values of the processed 462 spectral bands. There are five main blocks. The first block consists of a 1D convolution layer and a maximum pool layer, and then continues through a dropout layer with parameter 0.5. The second blockX contains three residuals module. The third blockX contains one downsampled module and three residuals module. The fourth blockX goes through one downsampled module and five residuals module before a dropout layer with parameter 0.5, and then continues through three residuals module. The fifth block consists of a mean pool layer and linear output layer. The number of convolution filters doubles as the block goes deeper (starting with 32 and ending with 128). All convolutional layers have a kernel size of 3 and a step size of 3. By connecting the convolutional layers together, deeper layers can be connected to a larger portion of the original input. Thus, different layers see the original input and learning ability at different levels. The last deeper layer outputs the SSC estimation, which converge to the ground truth value under the approximation of the MSE loss function.

#### 2.3.2. Machine Learning Models

In this work, we select four widely-used machine learning models as references to our deep learning based technique.As described in Table 1, they are Support Vactor Regression (SVR) (Castro-Neto et al., 2009), K-Nearest Neighbors Regression (KNNR) (Yao and Ruzzo, 2006), Adaptive Boosting Regression (AdaBoostR) (Freund et al., 1999), and Partial Least Squares Regression(PLSR) (Wold et al., 2001).

**Table 1:** Advantages, disadvantages, and applications of machine learning models in hyperspectrum.

**Machine learning models**	**Advantages**	**Disadvantages**	**Quality parameters**
SVR	Fast data fitting	Prone to overfitting phenomenon	Rice moisture (Sun et al., 2017)
KNNR	Low training time complexity	Computationally intensive	biomass (Tian et al., 2021)
Adaboost	Weak learners can be constantly updated	Vulnerable to noise interference	Soil organic matter (Wei et al., 2020)
PLSR	Suitable for data with multiple features	Not suitable for data with few features	SSC (Li et al., 2016)

#### 2.3.3. Experimental Setup

The algorithms are trained and run on a platform with an I7-8750H CPU and a 1,060 GPU. They are programmed using python and tensorflow etc. The datasets are divided as described in Table 2. The processed spectral data are used in the machine learning models while the raw spectral data are used in the Con1dResNet network. Since our deep learning model Con1dResNet can extract low to high dimensional features automatically, we use the original spectral data instead. We set Relu as the activation function, Adam as the optimizer, MSE as the loss function, the number of iterations to 50, and the batch size t o 16. After 50 iterations of training, the loss decreases from 72.86 at the beginning to 0.01, indicating a convergence for the algorithm.

**Table 2:** Cherry tomato SSC and firmness dataset partitioning.

**Sample size**	**Dataset**	**SSC (°Brix)**				**Firmness (** ***N*** **/cm** ^**2**^ **)**			
		**Max**	**Min**	**Mean**	**STD**	**Max**	**Min**	**Mean**	**STD**
Small	Total (50)	10.800	8.000	9.114	0.726	12.642	5.978	9.038	1.351
	Train set (35)	10.800	8.000	9.129	0.760	12.642	5.978	8.747	1.324
	Val set (5)	10.400	8.700	9.320	0.779	9.800	8.624	9.153	0.488
	Test set (10)	9.200	7.900	8.600	0.380	12.054	8.134	9.996	1.359
Large	Total(200)	11.100	7.200	8.719	0.662	12.936	5.978	8.853	1.229
	Train set (140)	11.100	7.200	8.790	0.726	12.936	5.978	9.140	1.266
	Val set (20)	9.200	7.800	8.500	0.407	9.996	7.305	8.345	0.708
	Test set (40)	9.000	7.200	8.455	0.478	10.192	7.056	8.102	0.858

## 3. Results

In this section, we evaluate our techniques in SSC and firmness estimation.

### 3.1. Hyperspectral Waveform Characteristics

**Figure 5:**
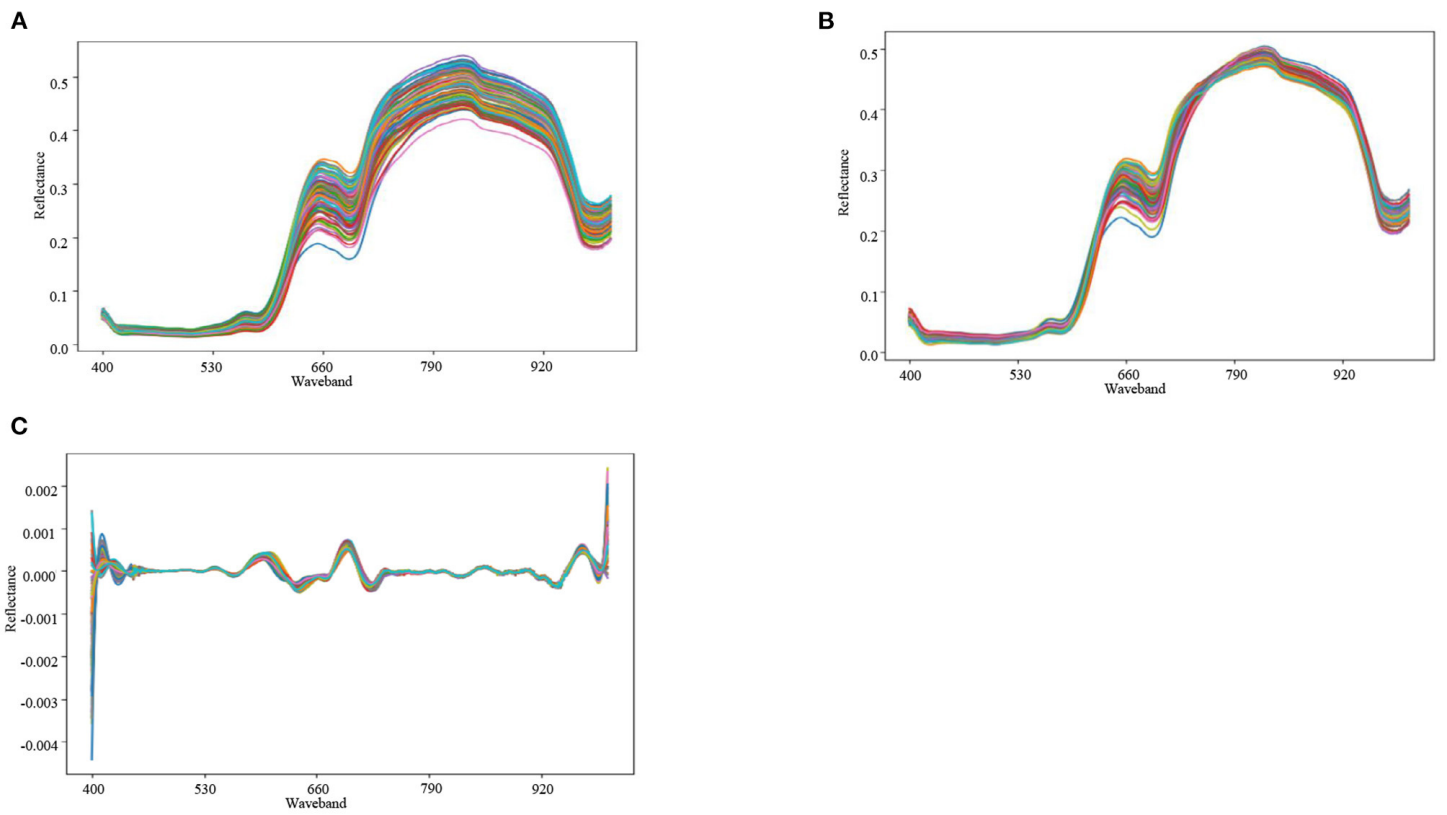
**(A)** Corrected spectral reflectance map. **(B)** MSC preprocessing. **(C)** Second-order differential preprocessing.

Figure 5A shows the reflectance spectra of 200 cherry tomato samples at 386–1,004 nm. The spectral trends are similar for each sample since the reflection substances are the same. The cherry tomatoes have a strong absorption band at 400–550 nm due to the presence of carotenoids in ripe tomatoes (Ecarnot et al., 2013). The reflectance data are then processed using MSC. As shown in Figure 5B, it can effectively reduce the noise and hence, smooth the curve. Finally, we use second order differentiation method (Ichige et al., 2006) to process the smoothed reflectance data and discover clear peaks at locations of 580–590, 680–690, and 970–980 nm, as shown in Figure 5C. The three peaks are likely to be attributed to the combined effect of the second overtone of OH key, water, and tomato surface color (Li et al., 2013; Qiu et al., 2018). Therefore, by proper processing, the variations in the spectral curves can reveal certain hidden information, such as SSC and water.

### 3.2. Analysis

Table 2 summarizes the distribution characteristics of SSC and firmness in different stages. The SSC and firmness measurements for the 50 and 200 samples are close to normally distributed around the mean values of 9.11° Brix, 9.04 N/cm^2^ and 8.72° Brix, 8.85 N/cm^2^, standard deviations (SD) of 0.76, 1.35 and 0.66, 1.23, respectively.

### 3.3. SSC Estimation Result

Four machine learning models are implemented and compared with our proposed Con1dResNet network. We use *R*^2^ and MSE as the evaluation metrics. They are calculated using the following equations.


$$R^{2}=1-\frac{\sum_{i}{\left(\hat{y_{i}}-y_{i}\right)}^{2}}{\sum_{i}{\left(\bar{y_{i}}-y_{i}\right)}^{2}}$$

$$MSE=\frac{1}{m}\overset{m}{\underset{i=1}{\sum }}{\left(y_{i}-\hat{y_{i}}\right)}^{2}$$

where $\hat{y_{i}}$ is the estimated value, *y*_*i*_ is the ground true value, and $\bar{y_{i}}$ is the ground true mean value. The optimal *R*^2^ and MSE values are 1 and 0, respectively.

**Figure 6:**
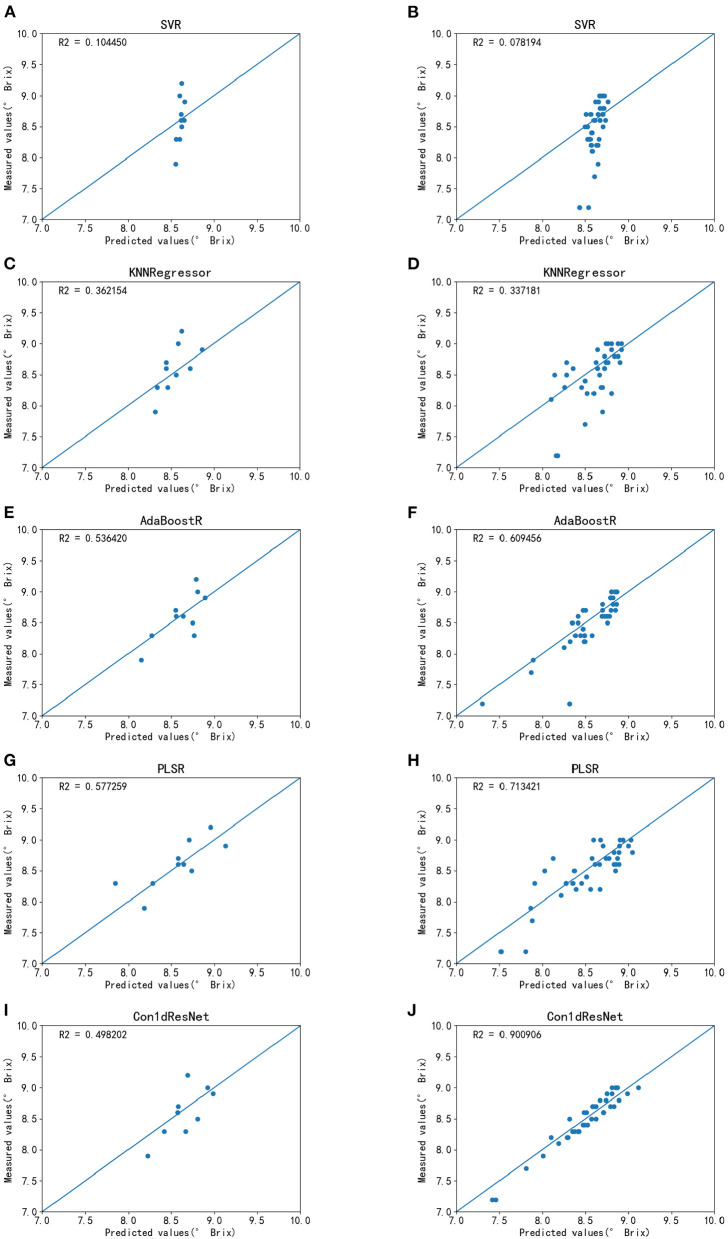
SSC estimation results for each model. **(A)** SVR estimation results on small sample data. **(B)** SVR estimation results on large sample data. **(C)** KNNR estimation results on small sample data. **(D)** KNNR estimation results on large sample data. **(E)** AdaBoostR estimation results on small sample data. **(F)** AdaBoostR estimation results on large sample data. **(G)** PLSR estimation results on small sample data. **(H)** PLSR estimation results on large sample data. **(I)** Con1dResNet estimation results on small sample data. **(J)** Con1dResNet estimation results on large sample data.

The experimental results are shown in Figure 6 and Table 3. In general, the second-order differential processing outperforms MSC. However, since the SVR and KNNR models lack the ability of data dimensionality reduction, the noise caused by unwanted reflectance cannot be removed. When the data size increases, the amount of interference also rises. Thus, the *R*^2^ value decreases as the data size increases. As expected, they have the worst performance with *R*^2^ < 0.4. For AdaBoostR, PLSR, and Con1dResNet models, *R*^2^ values increase with increasing datasets size. For a relatively smaller data size, the PLSR model achieves the best performance, with *R*^2^ of 0.577 and MSE of 0.055. As the data size increases, the performance of the Con1dResNet model is improved significantly, with *R*^2^ increasing from 0.498 to 0.901 (26.4% better than the second best) and MSE decreasing from 0.065 to 0.018.

**Table 3:** *R*^2^ and MSE of estimated SSC for each model.

**Sample size**	**Model**	**Preprocessed**	**Second-order differential**		**MSC**	
			***R* ^2^**	**MSE**	***R* ^2^**	**MSE**
Small (50)	SVR	✓	0.104	0.116	0.089	0.123
	KNNR	✓	0.362	0.083	0.289	0.096
	AdaBoost	✓	0.536	0.060	0.502	0.068
	PLSR	✓	0.557	0.055	0.528	0.062
	Con1dResNet	✗	*R* ^2^	0.498	MSE	0.065
Large (200)	SVR	✓	0.078	0.205	0.075	0.207
	KNNR	✓	0.337	0.147	0.316	0.152
	AdaBoost	✓	0.609	0.089	0.581	0.096
	PLSR	✓	0.713	0.064	0.710	0.067
	Con1dResNet	✗	*R* ^2^	0.901	MSE	0.018

### 3.4. Firmness Estimation Result

**Figure 7:**
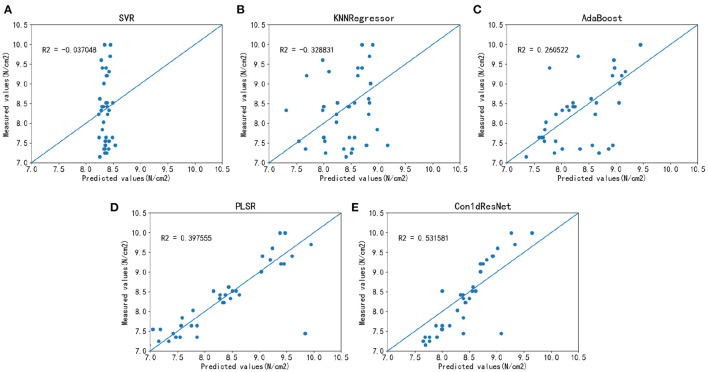
Estimation results of firmness for each model on a large sample dataset. **(A)** SVR estimation results on large sample data. **(B)** KNNR estimation results on large sample data. **(C)** AdaBoostR estimation results on large sample data. **(D)** PLSR estimation results on large sample data. **(E)** Con1dResNet estimation results on large sample data.

The same experimental setup is employed for firmness detection. As shown in Figure 7 and Table 4, when MSC is employed for AdaBoost and PLSR, their *R*^2^ values can be significantly improved (Wang et al., 2014). Therefore, we choose MSC as the preprocessing method for AdaBoost and PLSR, and second-order difference as the preprocessing method for SVR and KNNR. Although the method developed in this study has some advantages in data feature extraction compared with other methods, *R*^2^ is still only 0.53, which does not achieve the accurate estimation standard. The *R*^2^ of SVR and KNNR models is negative, which indicates the estimation accuracy is lower than the mean value.

**Table 4:** *R*^2^ and MSE of estimated SSC for each model with all sample.

**Model**	**Preprocessed**	**Second-order differential**		**MSC**	
		***R* ^2^**	**MSE**	***R* ^2^**	**MSE**
SVR	✓	−0.037	1.108	−0.054	1.116
KNNR	✓	−0.329	1.251	−0.456	1.318
AdaBoost	✓	0.217	0.694	0.261	0.675
PLSR	✓	0.384	0.552	0.398	0.548
Con1dResNet	✗	*R* ^2^	0.532	MSE	0.416

## 4. Discussion

The tomato flavor is important. SSC, which mainly consists of soluble sugars, can reflect the sweetness of cherry tomato. Hyperspectral imaging has been considered an effective technique for fruit SSC and firmness evaluation (Lu, 2004; Fan et al., 2015). In this work, we discover a great estimation result for SSC estimation, while an inferior result for firmness.

As shown in Table 3, our proposed method does not fit as well as PLSR and AdaBoost on small sample datasets. This is because Con1dResNet requires a large amount of data for training. When the amount of data is small, many models, especially for the deep learning based models, tends to become overfitting, which can significantly reduce the performance. However, for the PLSR model, it includes a principal component analysis component, which screen the band contribution first, and then selects 5–20 feature bands with relatively large contribution rates for regression. In that case, it can have a relatively good fit for small dataset samples. Moreover, AdaBoost constantly corrects the data with large fitting errors, and thus, achieve self-evolution. Thus, AdaBoost can also derive decent results in small dataset samples.

The extracted spectral (Guo C. et al., 2016) features can derive excellent estimation results for large sample size. The experimental results show that SVR and KNNR does not fit well on both the small and large sample data set. The performance of SVR and KNNR decrease when the data increase since few new “learning material” is generated for these two models when the data increases. In that case, the learning ability of the models can be more easily affected by the interference bands, which demonstrates that these two models are not suitable for SSC estimation.

As the number of sample size increases, our Con1dResNet model gradually outperforms other models due to the improved feature extraction ability of deep learning models (Dara and Tumma, 2018). Our model includes 34 layers of neurons, which can effectively extract rich data features. The residual learning structure can also help increasing the overall performance. Therefore, the accuracy of our method outperforms all the other methods for large-scale data samples. For applications with less samples, it is demonstrated that the accuracy of our technique is still relatively high. Moreover, our model is insensitive to anomalous data. It can be trained using pre-trained models and thus, reducing the training cost. The experimental results demonstrate that Con1dResNet can significantly outperform the existing machine learning based techniques, with *R*^2^ of 0.901 and MSE of 0.018. We believe that the experimental results of this work are also indicative for other horticultural crops.

For the hyperspectral images based tomato firmness, although it is reported that hyperspectral images can estimate fruit firmness (Lu, 2004; Fan et al., 2015), our experimental results suggest otherwise. Rahman et al. (2018) use PLSR to estimate tomato firmness using hyperspectral images in the 1,000–1,550 nm wavebands, and derive *R*^2^ value of 0.6724. It is a little higher than our experiment due to the differences in the used hyperspectral wavebands and the experimental environments. Therefore, in future work, for the estimation of firmness, we should explore a wider range of hyperspectral image wavebands, optimize the parameters for the firmness experiments, and improve the overall estimation accuracy.

## 5. Conclusion

In this work, we propose Con1dResNet, a deep learning based technique, to estimate the SSC and firmness of cherry tomatoes using hyperspectral images. With sufficient sample size, it can achieve better results than traditional machine learning methods. For SSC estimation, its *R*^2^ value is 0.901, which is 26.4% higher than PLSR, while its MSE is 0.018, which is 0.046 lower than PLSR. For Firmness estimation, its *R*^2^ value is 0.532, which is still 33.7% better than PLSR. The results indicate that hyperspectral imaging combined with deep learning can significantly improve the cherry tomato SSC and firmness estimation accuracies.

## Data Availability Statement

The raw data supporting the conclusions of this article will be made available by the authors, without undue reservation.

## Author Contributions

YX, QC, YC, QX, and ZS performed a conceptual and formal analysis of the study. QC, YX, ZC, and YC wrote the manuscript. YX, YC, QC, LZ, GZ, ZY, and QX designed the experiment. QC, LZ, and ZS wrote the experimental code. YX and QX verified the experimental results. All authors contributed to the article and reviewed the manuscript. All authors contributed to the article and approved the submitted version.

## Funding

This research was partially supported by National Key Research and Development Program of China (2018YFD1000800 and 2017YFE0114500), Key Research and Development Program of Zhejiang (2021C02052), National Natural Science Foundation of China (32172555), Zhejiang Provincial Agricultural (Vegetable) New Variety Breeding Project (2021C02065), China Agriculture Research System of MOF and MARA (CARS-23-G44), and State Key Laboratory Breeding Base for the Zhejiang Sustainable Pest and Disease Control (2010DS700124-ZZ1903).

## Conflict of Interest

The authors declare that the research was conducted in the absence of any commercial or financial relationships that could be construed as a potential conflict of interest.

## Publisher's Note

All claims expressed in this article are solely those of the authors and do not necessarily represent those of their affiliated organizations, or those of the publisher, the editors and the reviewers. Any product that may be evaluated in this article, or claim that may be made by its manufacturer, is not guaranteed or endorsed by the publisher.
